# SARS-CoV-2 induces “cytokine storm” hyperinflammatory responses in RA patients through pyroptosis

**DOI:** 10.3389/fimmu.2022.1058884

**Published:** 2022-12-01

**Authors:** Qingcong Zheng, Rongjie Lin, Yuchao Chen, Qi Lv, Jin Zhang, Jingbo Zhai, Weihong Xu, Wanming Wang

**Affiliations:** ^1^ Department of Orthopedics, 900th Hospital of Joint Logistics Support Force, Fuzhou, China; ^2^ Department of Paediatrics, Fujian Provincial Hospital South Branch, Fuzhou, China; ^3^ Department of Pharmacology and Toxicology, University of Mississippi Medical Center, Jackson, MS, United States; ^4^ Key Laboratory of Zoonose Prevention and Control at Universities of Inner Mongolia Autonomous Region, Medical College, Inner Mongolia Minzu University, Tongliao, China; ^5^ Department of Orthopedics, First Affiliated Hospital of Fujian Medical University, Fuzhou, China

**Keywords:** SARS-CoV-2, COVID-19, rheumatoid arthritis, pyroptosis, caspase-1, minocycline

## Abstract

**Background:**

The coronavirus disease (COVID-19) is a pandemic disease that threatens worldwide public health, and rheumatoid arthritis (RA) is the most common autoimmune disease. COVID-19 and RA are each strong risk factors for the other, but their molecular mechanisms are unclear. This study aims to investigate the biomarkers between COVID-19 and RA from the mechanism of pyroptosis and find effective disease-targeting drugs.

**Methods:**

We obtained the common gene shared by COVID-19, RA (GSE55235), and pyroptosis using bioinformatics analysis and then did the principal component analysis(PCA). The Co-genes were evaluated by Gene Ontology (GO), Kyoto Encyclopedia of Genes and Genomes (KEGG), and ClueGO for functional enrichment, the protein-protein interaction (PPI) network was built by STRING, and the k-means machine learning algorithm was employed for cluster analysis. Modular analysis utilizing Cytoscape to identify hub genes, functional enrichment analysis with Metascape and GeneMANIA, and NetworkAnalyst for gene-drug prediction. Network pharmacology analysis was performed to identify target drug-related genes intersecting with COVID-19, RA, and pyroptosis to acquire Co-hub genes and construct transcription factor (TF)-hub genes and miRNA-hub genes networks by NetworkAnalyst. The Co-hub genes were validated using GSE55457 and GSE93272 to acquire the Key gene, and their efficacy was assessed using receiver operating curves (ROC); SPEED2 was then used to determine the upstream pathway. Immune cell infiltration was analyzed using CIBERSORT and validated by the HPA database. Molecular docking, molecular dynamics simulation, and molecular mechanics-generalized born surface area (MM-GBSA) were used to explore and validate drug-gene relationships through computer-aided drug design.

**Results:**

COVID-19, RA, and pyroptosis-related genes were enriched in pyroptosis and pro-inflammatory pathways(the NOD-like receptor family pyrin domain containing 3 (NLRP3) inflammasome complex, death-inducing signaling complex, regulation of interleukin production), natural immune pathways (Network map of SARS-CoV-2 signaling pathway, activation of NLRP3 inflammasome by SARS-CoV-2) and COVID-19-and RA-related cytokine storm pathways (IL, nuclear factor-kappa B (NF-κB), TNF signaling pathway and regulation of cytokine-mediated signaling). Of these, CASP1 is the most involved pathway and is closely related to minocycline. YY1, hsa-mir-429, and hsa-mir-34a-5p play an important role in the expression of CASP1. Monocytes are high-caspase-1-expressing sentinel cells. Minocycline can generate a highly stable state for biochemical activity by docking closely with the active region of caspase-1.

**Conclusions:**

Caspase-1 is a common biomarker for COVID-19, RA, and pyroptosis, and it may be an important mediator of the excessive inflammatory response induced by SARS-CoV-2 in RA patients through pyroptosis. Minocycline may counteract cytokine storm inflammation in patients with COVID-19 combined with RA by inhibiting caspase-1 expression.

## Introduction

In 2019, SARS-CoV-2-caused COVID-19 was recognized as a public health emergency of international concern (PHIEC) and subsequently identified as a pandemic by the World Health Organization (WHO) (1–6). SARS-CoV-2 is the third widespread coronavirus outbreak after SARS CoV in 2003 (7, 8) and MERS CoV in 2012 (9, 10). Droplets and aerosols mostly transmit SARS-CoV-2 at close range (11–13). From the COVID-19 dashboard of the Johns Hopkins Coronavirus Resource Center: As of 2022.8.28, more than 200 countries/regions worldwide have recorded over 600 million confirmed cases and over 6.48 million deaths, with a total of 12.124 billion vaccine doses administered (14). Coronaviruses (CoVs) are a group of enveloped viruses with a single-stranded RNA genome (+ssRNA) that exhibits a high mutation rate and variable recombination rates (15–17). SARS-CoV-2 is the ninth coronavirus threatening human health (18, 19) and has a high degree of host genetic variation (20–23). SARS-CoV-2 can encode 29 proteins (24, 25), consisting of 16 non-structural proteins (NSP) (26), 4 structural proteins (spike [S], envelope [E], membrane [M], and nucleocapsid [N]) (27), and 9 auxiliary proteins (28). COVID-19 is not just a respiratory disease but also a systemic disease that affects many of the body’s systems and organs (29, 30). SARS-CoV-2 infection frequently disrupts the immune system (31), resulting in increased expression of autoantigens during infection and the development of autoantibodies due to the organism’s potential antigenic cross-reactivity (32–34). SARS-CoV-2 is not only predisposed to the onset and progression of autoimmune diseases (35–37), but even SARS-CoV-2 vaccination can trigger autoimmune phenomena (38, 39). Consequently, patients with autoimmune illnesses have a higher risk of contracting COVID-19 (40, 41).

The COVID-19 Global Rheumatology Alliance Global Registry records: As of 2022.08.31, the most common autoimmune/rheumatic disease among COVID-19 patients is RA (40.92%) (42). RA is one of the most prevalent autoimmune diseases, with a prevalence of up to 1 percent (43–46), and its expanding population coverage has posed a significant threat to global public health (47). The three primary causes of RA development are genetic, environmental, and immunological factors (48, 49), with viruses, as part of the environmental factors, playing a significant role in the development of RA (50, 51). Correspondingly, the immunological dysregulation in RA patients favors the invasion of SARS-CoV-2 (52, 53). Additionally, the traditional use of DMARDs and glucocorticoids in RA enhances viral replication *via* immunosuppression, and the use of biological agents (e.g., TNF-α-inhibitors) also raises the likelihood of viral infection in RA (54–57). Therefore, there may be a potential mutual pathogenic factor between COVID-19 and RA that contributes to disease progression, and we need to find appropriate therapeutic agents to combat it.

Pyroptosis is an emerging form of regulated cell death (RCD) and an active area of research (58). It is caused by innate immune dysregulation and disruption of organism/cellular homeostasis due to pathogen invasion (59), as shown by increased plasma membrane permeability, cell swelling, and rupture (60, 61). caspase-1 is one of the first pro-pyroptosis inflammatory cystathiases identified (62–65), creating NLRP3 inflammasome by binding to NLRP3, apoptosis-associated speck-like Protein (ASC), which establishes the canonical route of pyroptosis leading to cell lysis and the release of IL-1β and IL-18 (66–70). Firstly, active NLRP3 inflammasome and caspase-1 are detected in the peripheral blood and tissues of COVID-19 patients and are positively correlated with severity markers for COVID-19 (e.g., IL-6) (71). In SARS-CoV-2 infected cells, NLRP3 inflammasome and caspase-1 activity increase and promote pyroptosis and cytokine storm (72–74). Secondly, the overactivation of NLRP3 inflammasome and caspase-1 in individuals with RA’s serum, synovium, and synovial fluid induces pyroptosis and inflammatory responses and is positively linked with disease activity (75–79). Thus, the caspase-1-mediated classical pyroptosis pathway may be an important cause of the vicious cycle of cytokine storm caused by the interaction between COVID-19 and RA disease. This study investigates the pathogenesis and disease targets of COVID-19 associated with RA through bioinformatics and network pharmacology analysis as well as computer-aided drug design methods and explores the drug and pharmacology of this target.

## Methods

### Data collection and processing

Three RA datasets (GSE55235, GSE55457, GSE93272) were screened using the National Center for Biotechnology Information (NCBI) Gene Expression Omnibus (GEO) (https://www.ncbi.nlm.nih.gov/geo/) (**Table 1**). GSE55235 contains synovial tissue samples from 10 RA cases and 10 healthy people. GSE55457 contains synovial tissue samples from 13 RA cases and 10 healthy people, and GSE93272 contains 232 whole blood samples from RA patients and 43 healthy people. The GeneCards database (https://www.genecards.org/) (80) platform searched for the keywords “SARS-CoV-2” and “COVID-19” and found 4055 and 4778 related genes. Xiong et al., 2020 (81), Ziegler et al., 2020 (82), and Jain et al., 2020 (83), respectively, contributed an additional 25, 17, and 28 COVID-19-related genes (**Supplementary Table 1**). A total of 5103 COVID-19-related genes were obtained by pooling and de-duplicating these genes. Similarly, a search of the GeneCards database using the keyword “pyroptosis” yielded 254 related genes.

**Table 1:** Basic information of selected datasets.

Dataset ID	Platform	Tissue(Homo sapiens)	Experimental group	Normal control	Experiment type
GSE55235	GPL96	Synovium	10	10	Array
GSE55457	GPL96	Synovium	13	10	Array
GSE93272	GPL570	Whole blood	232	43	Array

### Identification of co-genes

The empirical Bayesian method in the limma package (http://www.bioconductor.org/packages/release/bioc/html/limma.html) (84) was used to analyze the RA and healthy controls (HC) groups of the GSE55235 dataset in different gene expression analyses. |log2 FC| >0.5 and *P*< 0.05 as the cutoff. Further mapping of volcanoes using the ggplot2 package to reflect RA-differentially expressed genes (DEGs). Co-genes were obtained from the intersection of COVID-19, RA-DEGs (GSE55235), and pyroptosis-related genes using the Venn-diagram package in R software and subjected to PCA.

### GO, KEGG, and ClueGO enrichment analyses of co-genes

For the investigation of the pathway and function of the Co-genes, the R package “clusterProfiler” (85) was used to conduct GO and KEGG enrichment analyses. Co-genes are visualized through ClueGO (a plug-in for Cytoscape, using kappa’s statistical analysis method) to differentiate between up-and down-regulated genes to construct interactive gene network maps and analyze the function of target gene sets.

### PPI network analysis and machine learning for the identification of hub genes

The STRING database (https://string-db.org/) (86) was utilized to analyze the Co-genes and build a PPI network with a confidence score > 0.40 as the threshold. The k-means algorithm is an effective unsupervised machine learning technique (87). It enables the prediction of protein-protein interactions without explicit data labeling. We used the k-means algorithm (the network was clustered to a specified number of clusters, the number clusters: 3) Clustering analysis of Co-genes. The Cytoscape platform (88) is utilized to visualize PPI network data, while the MCODE (a Cytoscape plug-in) is utilized for modular analysis of PPI networks. The cytoHubba uses the Degree algorithm to identify Hub genes from Co-genes.

### Metascape, geneMANIA and network analyst analyses of hub genes

Metascape (https://metascape.org/gp/index.html#/main/step1) (89) is a gene function analysis website that aggregates over 40 databases and groups genes into clusters based on Terms with a *P<* 0.01, a minimum count of 3, and an enrichment factor >1.5 to group genes into clusters and find pathways for the enrichment of Hub genes and associated functional annotations. Cytoscape connected terms with similarity > 0.30 to further build a network graphic to capture the linkages between gene clusters. GeneMANIA (http://www.genemania.org) (90) is a website that integrates different databases and technologies, including Gene Expression Omnibus (GEO) and the Biological General Repository for Interaction Datasets (BioGRID), for predicting the functions of Hub genes and identifying gene priority and interconnections. NetworkAnalyst (https://www.networkanalyst.ca/) (91) is a website for visual analysis of gene expression profiling and meta-analysis. The hub genes were analyzed for associations with potentially relevant medications (DrugBank Version 5.0) by the site’s Protein-drug interactions function (minimum network).

### Screening for minocycline-related target genes and co-hub genes

CASP1, CASP3, and ILB in the hub genes were closely related to minocycline from NetworkAnalyst analysis. Therefore, minocycline was hypothesized to be an effective drug against this mechanism, and relevant validation was carried out. We used SwissTargetPrediction (http://www.swisstargetprediction.ch/) (92), CTD (http://ctdbase.org/) (93), Drugbank (https://go.drugbank.com/drugs/DB01017) (94) and STITCH (http://stitch.embl.de/cgi/input.pl) which are four databases to search for potentially related genes of minocycline. The STITCH database unifies drug-gene connections between more than 68,000 distinct compounds and 1.5 million genes; we utilize STITCH to visualize minocycline and target genes. COVID-19, RA-DEGs (GSE55235), pyroptosis-related genes, and minocycline-related target genes were intersected to determine the set of Co-targets. Subsequently, the Hub genes were intersected with the Co-targets to obtain Co-hub genes.

### Establishment of the TF-hub genes and miRNA-hub genes network

Co-hub genes were submitted to the NetworkAnalyst platform, TFs were obtained from the ENCODE database, and miRNAs were obtained from miRTarBase and TarBase. Visualization of TF-hub genes and miRNA-hub genes network using Cytoscape.

### Validation of co-hub genes and identification of key gene

To increase the reliability of the results as well as comprehensiveness, we included GSE55457 and GSE93272 as validation sets in this study. The intersection of the co-hub genes, RA-DEGs (GSE55457) and RA-DEGs (GSE93272), was identified as a key gene. Boxplot analyzed the expression of the key gene, and ROC (95) was used to determine the sensitivity and specificity of the key gene. The area under the curve (AUC) > 0.8 is considered to have a significant diagnostic value.

### Upstream pathway activity

SPEED2 (https://speed2.sys-bio.net/) (96) is an upstream signaling pathway enrichment analysis platform that evaluates the significance of 16 classical signaling pathways based on *P*-values using gene set data from human cell biology research. We used the bates test in SPEED2 to predict the upstream signaling pathways of the co-hub genes and the Key gene.

### Analysis of immune cell infiltration

The CIBERSORT algorithm (http://CIBERSORT.stanford.edu/) is a linear support vector regression-based methodology (97) applied to assess the makeup and number of immune cells in RA and HC. The relationship between the expression of the key gene and the abundance of immune cells in RA was revealed using person correlation coefficient analysis to find the immune cells closely related to it. The Human Protein Atlas (https://www.proteinatlas.org/) contains data on the tissue and cellular distribution and expression abundance of nearly all human proteins. The HPA database was utilized to validate the key gene-immune cell associations to guarantee the accuracy of the results.

### Molecular docking

Molecular docking techniques were used to verify the affinity of minocycline to the crystal structure of the protein expressed by the Key gene. First, a two-dimensional (2D) structure of minocycline was obtained in sdf format from the Drugbank database or the PubChem database (https://pubchem.ncbi.nih.gov/) (98) for use as a ligand. Entry for Key gene obtained from Uniport database (https://www.uniprot.org/) (99) (CASP1: P29466). Enter the entry into the RCSB PDB database (https://www.rcsb.org/) (100) and download the protein structure in pdb format to use as a receptor. Second, using ChemBio 3D Ultra 12.0 software, the 2D structure of the ligand (minocycline) was transformed to a 3D structure, optimized, and saved in mol2 format. The receptor (caspase-1) was processed using PyMOL 2.4.0 software to remove solvent molecules and ligands and then saved in pdb format. Third, After processing the ligands and receptors in Autodock 1.5.6 software and saving the results in pdbqt format, molecular docking was used to identify the activity pockets of candidate loci and export the results in gpf format. Finally, the AutoDock Vina software was used to carry out the molecular docking commands, and PyMOL 2.4.0 was used to visualize and analyze the results.

### Molecular dynamics simulation and molecular mechanics-generalized born solvent accessibility

Further investigation of the dynamic properties, stability, and structural flexibility of protein-drug complexes can be done by molecular dynamics simulations. It permits the examination of the interaction between the drug and the amino acid residues of the target protein and acts as an in-depth validation of molecular docking. MD to MDS and MM-GBSA calculations are a series of workflows for computer-aided drug design to study the properties of ligand-receptor interactions.

AMBER 18 was used to examine the stability of the complexes by simulating the molecular docking of ligands and receptors using all-atom MDS of ligands and receptors. Before the simulation, the charge of the minocycline was determined using the HF-SCF (6-31G**) computation with the antechamber module and gauss 09 software. The GAFF2 small molecule force field and the ff14SB protein force field were utilized to describe, respectively, the ligand (minocycline) and the receptor (caspase-1) (101, 102). The LEaP module was utilized to introduce hydrogen atoms, and a TIP3P solvent cartridge was added at 10Å. The system’s charge is then balanced by adding Na^+^/Cl^-^, and the topology and parameter files required for the molecular simulation are then output. Optimization of system energy *via* a 2500-step steepest descent method and a 2500-step conjugate gradient method. The system was warmed up at 200 ps and stabilized from 0 K to 298.15 K, followed by a 500 ps NVT ensemble simulation and a 500 ps equilibrium simulation. The system was warmed up at 200 ps, from 0 K to 298.15 K, followed by an NVT system simulation (isothermal isomer) at 500 ps, followed by an equilibrium simulation (isothermal isobaric) at 500 ps. The final NVT system simulation (isothermal isobaric) was carried out for 100 ns. Other parameters: truncation distance set to 10 Å, collision frequency γ set to 2 ps^-1^, system pressure 1 atm, integration step 2 fs, trajectory saved at 10 ps intervals.

The free energy of binding between receptor and ligand is calculated by the MM/GBSA method (103, 104). The specific formula is as follows:


$$\text{Δ}G_{bind}={\text{ΔG}}_{\text{complex}}\;–\;\left({\text{ΔG}}_{\text{receptor}}+{\text{ ΔG}}_{\text{ligand}}\right)$$

$$={\text{ΔE}}_{\text{internal}}+{\text{ΔE}}_{\text{VDW}}+{\text{ΔE}}_{\text{elec}}+{\text{ΔG}}_{\text{GB}}+{\text{ΔG}}_{\text{SA}}$$

ΔE_internal_ : Internal energy, ΔE_VDW_ : Van der Waals interactions, ΔE_elec_ : Electrostatic interactions, ΔG_GB_ and ΔG_SA_ : solvation-free energy.

**Figure 1:**
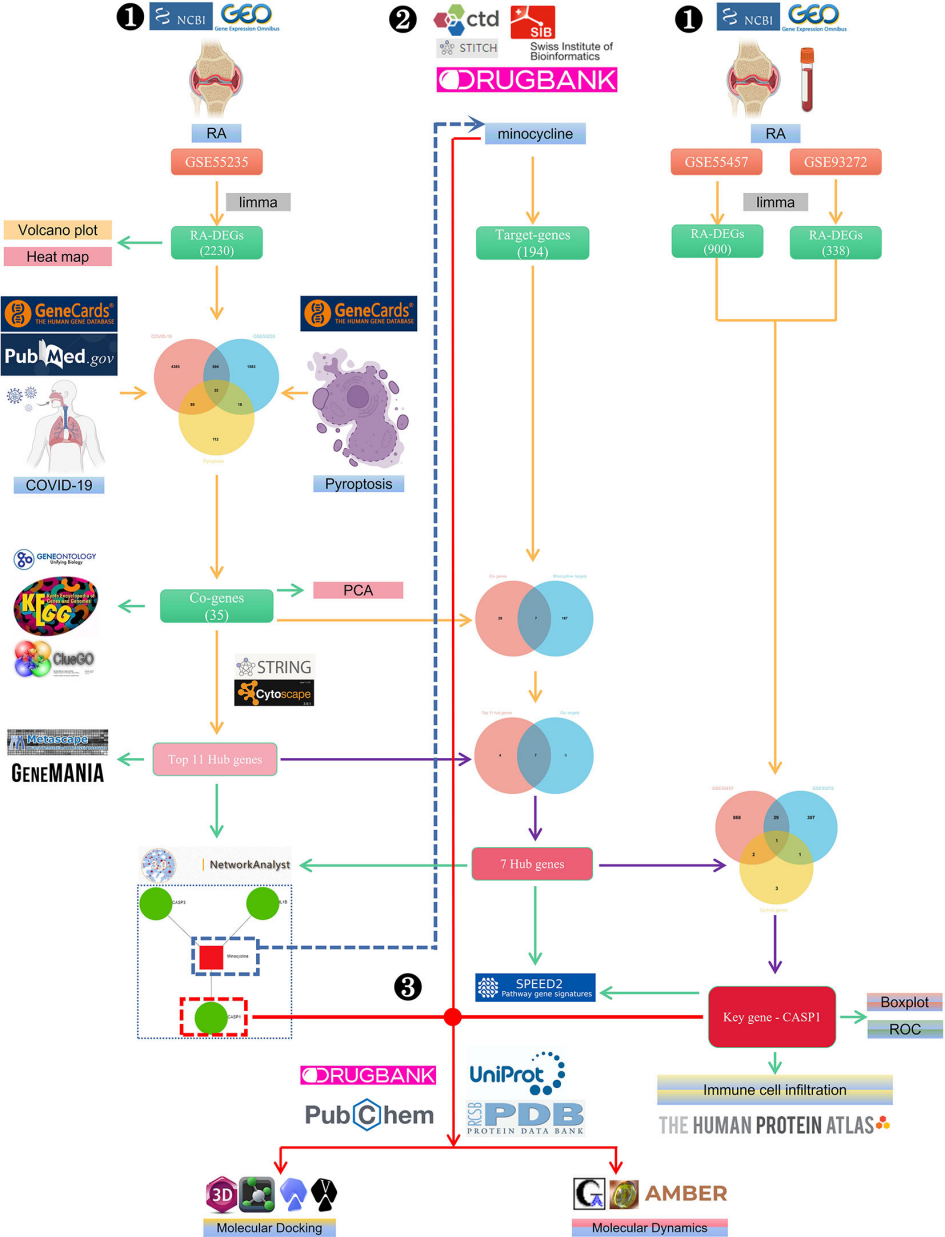
The schematic block diagram of the entire workflow of this study. ❶ Bioinformatics analysis. ❷ Network pharmacology. ❸ Computer-aided drug design.

The flowchart shows all of our study’s key and important procedures (**Figure 1**). The GitHub page for this study is HTTPS(https://github.com/zheng5862/COVID-19-RA.git).

## Results

### Identification of co-genes

**Figure 2:**
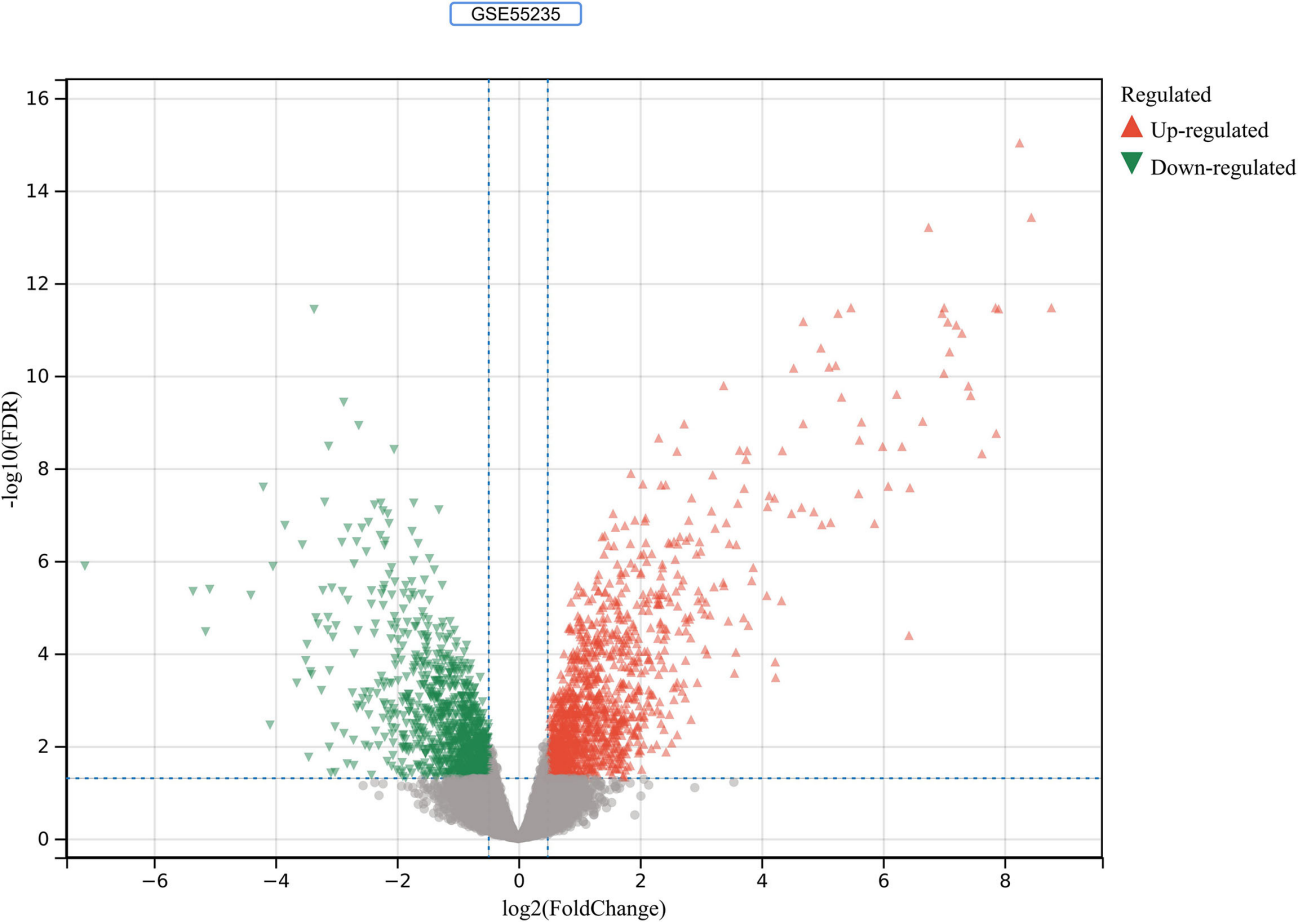
RA-DEGs identification. In the GSE55235 dataset, red triangles represent upregulated genes (*P* < 0.05), green triangles represent downregulated genes (*P* < 0.05), and gray dots represent genes not significantly differentially expressed across the RA and HC groups (*P* > 0.05).

**Figure 3:**
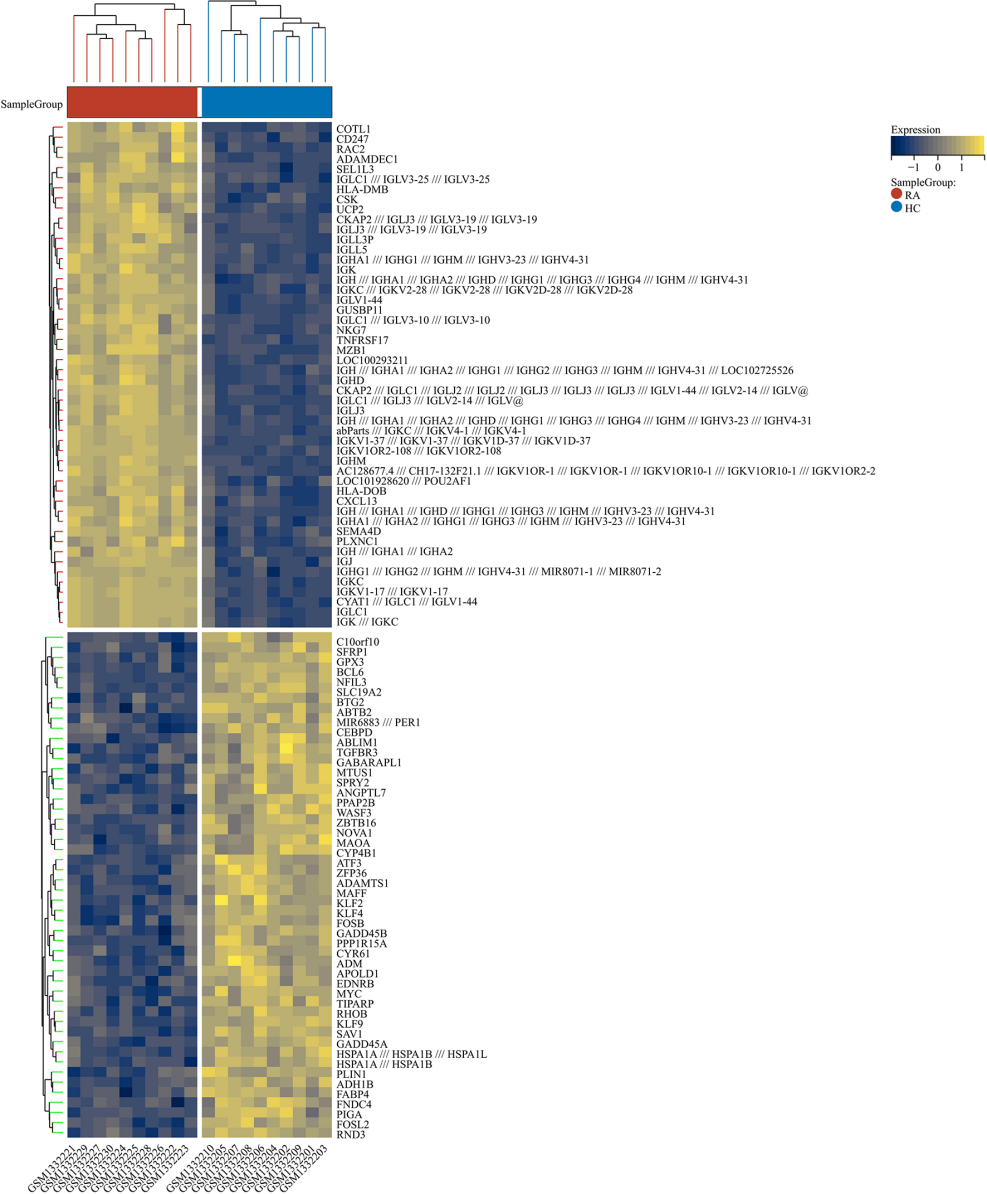
RA-DEGs distribution. The Clustering heat map displays the top one hundred DEGs from the GSE55235 dataset. The samples from the RA group were colored red, while those from the HC group were colored blue. Yellow rectangles represent highly expressed genes (*P* < 0.05), while blue rectangles represent lowly expressed genes (*P* < 0.05).

**Figure 4:**
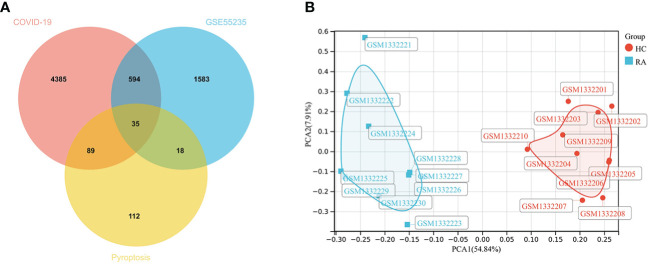
Screening Co-genes. **(A)** Venn-diagram on COVID-19, RA-DEGs (GSE55235), pyroptosis-related genes. Co-genes include 35 genes. **(B)** PCA analysis of Co-genes in the GSE55235 dataset: PC1 (54.84%) and PC2 (7.91%).

2230 RA-DEGs were obtained from the GSE55235 dataset and visualized using volcano maps and clustered heat maps (**Figures 2**, **3**). Co-genes are intersecting genes for COVID-19, RA-DEGs (GSE55235), and pyroptosis and include 35 genes, of which 23 are upregulated and 12 are down-regulated (**Figure 4A**). PCA analysis of the Co-genes in the GSE55235 dataset revealed that PC1 (54.84%) and PC2 (7.91%) confirmed the Co-genes’ significant reliability and between-group variability (**Figure 4B**).

### Functional enrichment analyses of co-genes

**Figure 5:**
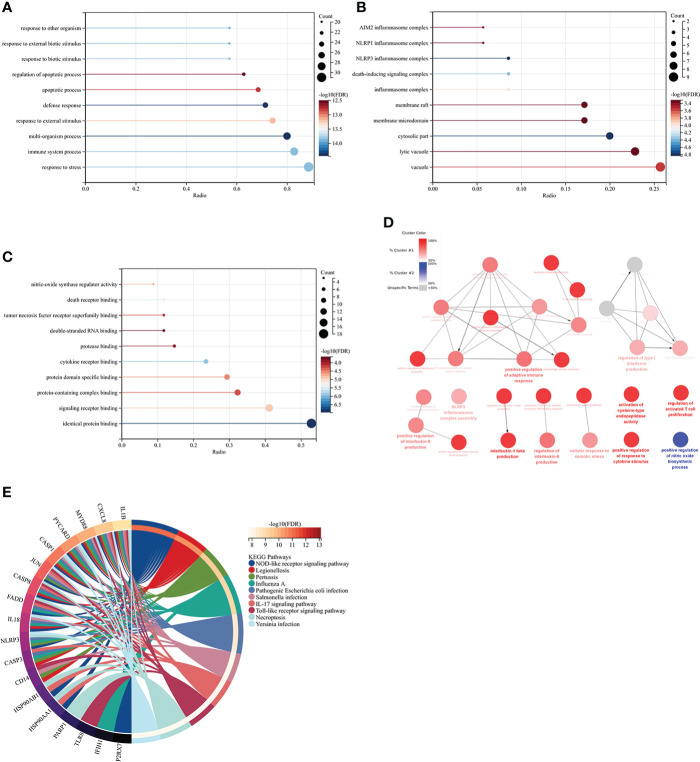
Co-genes functional enrichment analysis using GO, ClueGO, and KEGG. **(A)** Enrichment of Co-genes in BP. **(B)** Enrichment of Co-genes in CC. **(C)** Enrichment of Co-genes in MF. **(D)** Co-genes Analysis Using ClueGO. Red-denoted pathways for upregulated genes, while blue-denoted pathways for downregulated genes. **(E)** Co-genes Analysis Using KEGG.

GO analysis showed that the biological process (BP) was mainly enriched in the immune system process (**Figure 5A**). Cellular component (CC) was mainly enriched in the cytoplasm, inflammasome complex, death-inducing signaling complex, NLRP3, and NLRP1 inflammasome complex (**Figure 5B**). Molecular function (MF) was mainly enriched in signaling receptor binding, protein domain-specific binding, cytokine receptor binding, tumor necrosis factor receptor superfamily binding, and death receptor binding (**Figure 5C**). The ClueGO analysis showed visually that the upregulated genes of Co-genes were mainly enriched in NLRP3 inflammasome complex, positive response to cytokine stimulus, cytokine production involved in immune response, and regulation of interleukin (IL-1β, IL-6, IL-8, IL-17) production (**Figure 5D**). KEGG analysis was mainly enriched in the NOD-like receptor (NLR) signaling pathway, the IL-17 signaling pathway, and the Toll-like receptor (TLR) signaling pathway (**Figure 5E**).

### PPI network analysis and machine learning for hub genes

**Figure 6:**
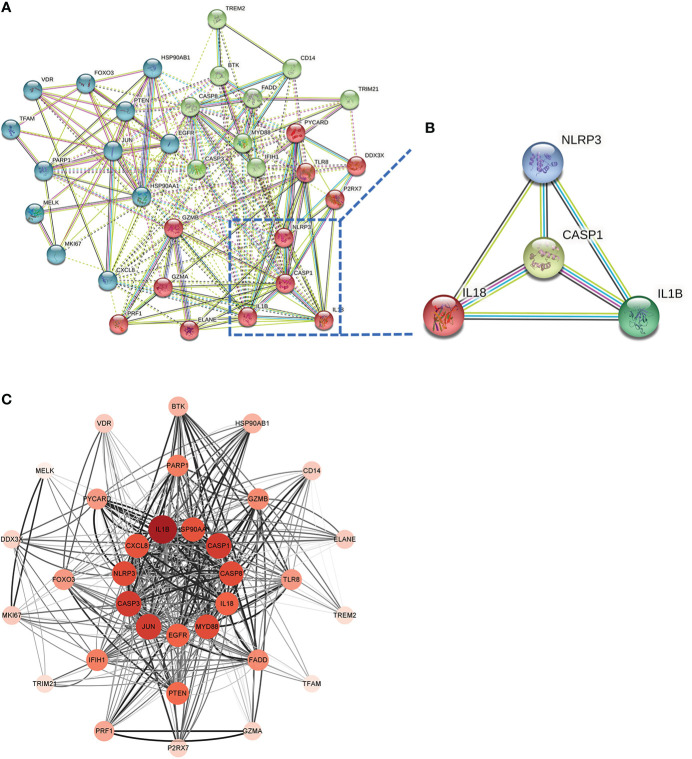
Screening Hub genes. **(A)** PPI network diagram obtained after applying the k-means algorithm based on machine learning to the Co-genes. The four genes in the lower right-hand corner of the enlarged diagram are CASP1, NLRP3, IL1B, and IL18. **(B)** PPI network diagram after processing with Cytoscape software. **(C)** The Top 11 hub genes are filtered using the Degree algorithm under the CytoHubba package condition.

This PPI network has 35 nodes, 202 edges, an average node degree of 11.5, and an average local clustering coefficient of 0.632 (**Figure 6A**). Using a machine learning algorithm, the k-means clustering analysis of the PPI data predicted the four genes in the lower right corner of the amplified content to be CASP1, NLRP3, IL1B, and IL18 (**Figure 6B**). These are the genes for the four most important proteins in the caspase-1-driven classical pyroptosis pathway. By using the degree algorithm of the CytoHubba program to the PPI data, the distribution of genes becomes specific and hierarchical, and it can be seen that the top 11 hub genes in the center of the ring were: IL1B, CASP1, CASP3, JUN, MYD88, CASP8, NLRP3, HSP90AA1, CXCL8, IL18, EGFR (where the Degree algorithm values for IL18 and EGFR were equal) (**Figure 6C**).

### Functional network analysis of the top 11 hub genes

**Figure 7:**
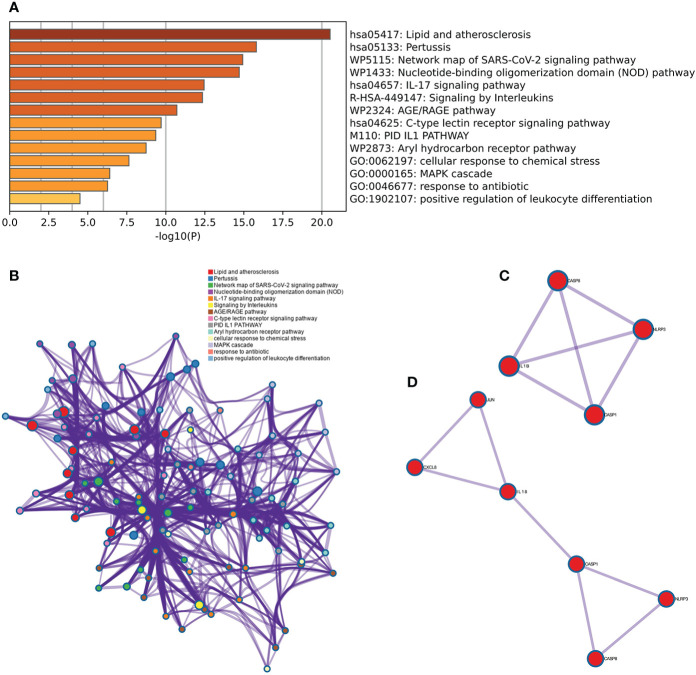
Metascape analysis of Hub genes. **(A)** Pathway and process richness analysis. **(B)** The network is shown using Cytoscape^5^, with nodes with the same cluster ID typically located close to one another. **(C, D)** Protein-protein Interaction Enrichment Analysis.

**Figure 8:**
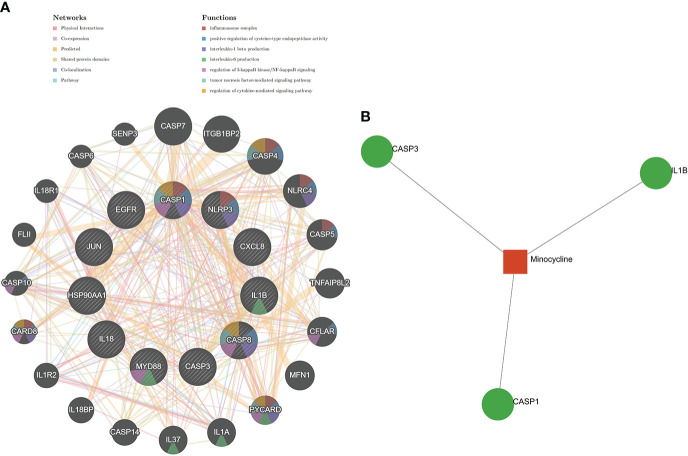
GeneMANIA and NetworkAnalyst analysis of Hub genes. **(A)** The GeneMANIA database examined the gene-gene interaction network of the top 11 hub genes and the 20 most nearby genes. Each node represents a gene. The color of the node links shows the relationship between the relevant genes. **(B)** Results for the top 11 Hub genes by NetworkAnalyst’s Protein-Drug Interaction Function (DrugBank database 5.0). Drugs were in red and target genes were in green.

The results of the Metascape analysis were as follows. In pathway and process enrichment analysis, the main enrichments were in the network map of the SARS-CoV-2 signaling pathway; Nucleotide-binding oligomerization domain (NOD) pathway; and Signaling by Interleukins (**Table 2**) (**Figure 7A**). Network diagrams will allow visualization of the associations between the pathways (**Figure 7B**). In the PPI enrichment analysis, the main enrichments were in the NOD pathway, the activation of the NLRP3 inflammasome by SARS-CoV-2 (**Figure 7C**), and the NLR signaling pathway (**Figure 7D**). Inflammasome complex, positive regulation of cysteine-type endopeptidase activity, production of IL(LI-1β, IL-6), NF-κB signaling, TNF-mediated signaling pathway, and regulation of cytokine-mediated signaling pathway were all enriched in GeneMANIA analysis of the top 11 hub genes (**Figure 8A**). Of these, CASP1 is the most involved in the pathway. The protein-drug interactions function on NetworkAnalyst (DrugBank database 5.0) found minocycline to be closely related to CASP1, CASP3, and IL1B (**Figure 8B**).

**Table 2:** Pathway and Process Enrichment Analysis in metascape.

GO	Category	Description	Count	%	Log10(P)	Log10(q)
hsa05417	KEGG Pathway	Lipid and atherosclerosis	10	90.91	-20.53	-16.18
hsa05133	KEGG Pathway	Pertussis	7	63.64	-15.8	-12.3
WP5115	WikiPathways	Network map of SARS-CoV-2 signaling pathway	8	72.73	-14.93	-11.49
WP1433	WikiPathways	Nucleotide-binding oligomerization domain (NOD) pathway	6	54.55	-14.71	-11.31
hsa04657	KEGG Pathway	IL-17 signaling pathway	6	54.55	-12.45	-9.28
R-HSA-449147	Reactome Gene Sets	Signaling by Interleukins	8	72.73	-12.35	-9.22
WP2324	WikiPathways	AGE/RAGE pathway	5	45.45	-10.71	-7.68
hsa04625	KEGG Pathway	C-type lectin receptor signaling pathway	5	45.45	-9.7	-6.91
M110	Canonical Pathways	PID IL1 PATHWAY	4	36.36	-9.36	-6.59
WP2873	WikiPathways	Aryl hydrocarbon receptor pathway	4	36.36	-8.74	-6.09
GO:0062197	GO Biological Processes	cellular response to chemical stress	5	45.45	-7.64	-5.13
GO:0000165	GO Biological Processes	MAPK cascade	4	36.36	-6.41	-4.08
GO:0046677	GO Biological Processes	response to antibiotic	3	27.27	-6.27	-3.96
GO:1902107	GO Biological Processes	positive regulation of leukocyte differentiation	3	27.27	-4.5	-2.49

### Identification of minocycline-related target genes and co-hub genes

**Figure 9:**
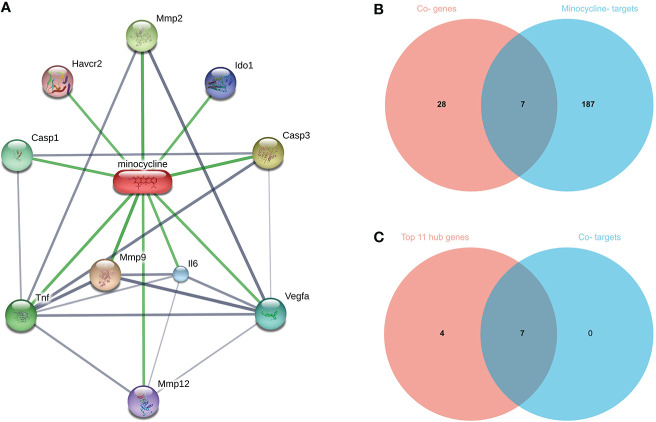
**(A)** Network diagram of minocycline and related target genes on the STITCH platform, with minocycline in capsules and related target genes in circles. **(B)** Venn-diagram of Co-genes versus minocycline-targets. **(C)** Venn diagram of the top 11 hub genes versus Co-targets, with Co-targets all contained in the top 11 hub genes.

Top 100, 92, 12, and 10 minocycline-related target genes from SwissTargetPrediction, CTD, Drugbank, and STITCH databases, respectively (**Supplementary Table 2**). We can visualize the connection between minocycline, each target gene, and gene to gene in the STITCH interaction network diagram (**Figure 9A**). A total of 194 minocycline-related Targets were obtained by pooling the total genes and removing duplicates. Co-targets were 194 genes intersecting with COVID-19, RA-DEGs (GSE55235), and pyroptosis-related genes, including 7 genes: CASP1, CASP8, IL1B, CASP3, JUN, EGFR, CXCL8 (**Figure 9B**). Co-targets were intersected with the top 11 hub genes to obtain the Co-hub genes (**Figure 9C**). All 7 genes in the Co- Targets were contained in the top 11 hub genes, suggesting that the targets of minocycline action may be proteins of core genes involved in the pyroptosis mechanism of COVID-19 and RA.

### TF-hub genes and miRNA-hub genes network for co-hub genes

**Figure 10:**
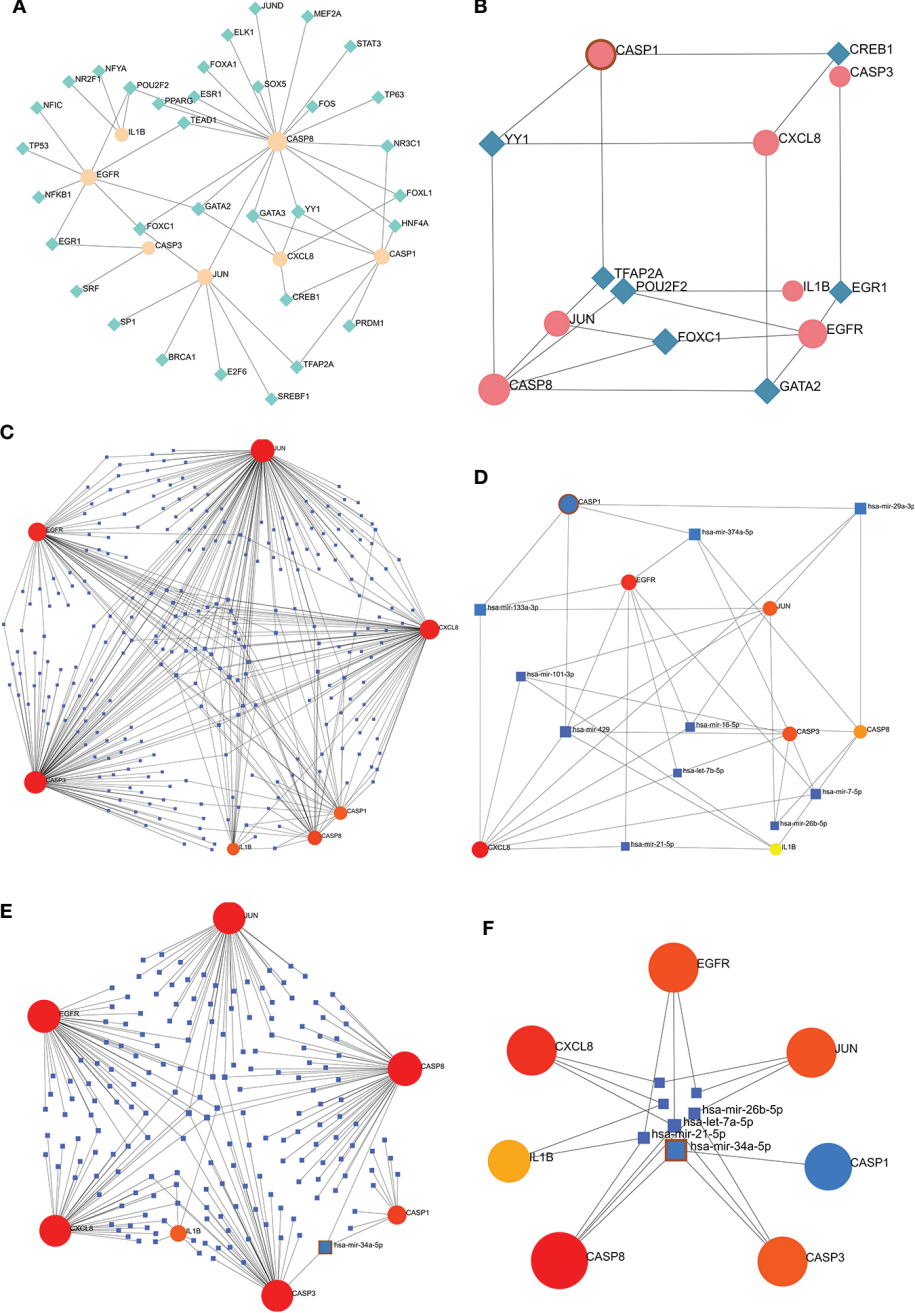
TF-hub genes and miRNA-hub genes network construction using NetworkAnalyst. **(A, B)** TF-hub genes network and simplified diagram. Circles were genes, while squares were TFs. **(C, D)** miRNA-hub genes network and simplified diagram (TarBase version 8.0). Circles represent genes, while squares are miRNAs. **(E, F)** miRNA-hub genes network and simplified diagram (miRTarBase v8.0). Circles represent genes, while squares are miRNAs.

The TF-hub genes network consists of 7 seeds, 51 edges, and 40 nodes (**Figure 10A**), and the simplified minimum network consists of 7 seeds, 19 edges, and 14 nodes (**Figure 10B**). YY1 has the potential to regulate CASP1, CASP8, and CXCL8. The miRNA-hub genes analyzed using the TarBase package consisted of 7 seeds, 407 edges, and 267 nodes (**Figure 10C**), and the simplified minimum network consisted of 7 seeds, 40 edges, and 17 nodes (**Figure 10D**). CASP1, CASP3, IL1B, CXCL8, and JUN were all closely related to hsa-mir-429. The miRNA-hub genes analyzed using the miRTarBase package consisted of 7 seeds, 210 edges, and 189 nodes (**Figure 10E**), and the simplified minimum network consisted of 7 seeds, 19 edges, and 14 nodes (**Figure 10F**). CASP1, CASP3, and CASP8 were all closely related to hsa-mir-34a-5p. In conclusion, YY1, hsa-mir-429, and hsa-mir-34a-5p may play an important role in the expression of CASP1.

### Validation of co-hub genes and identification of key gene

**Figure 11:**
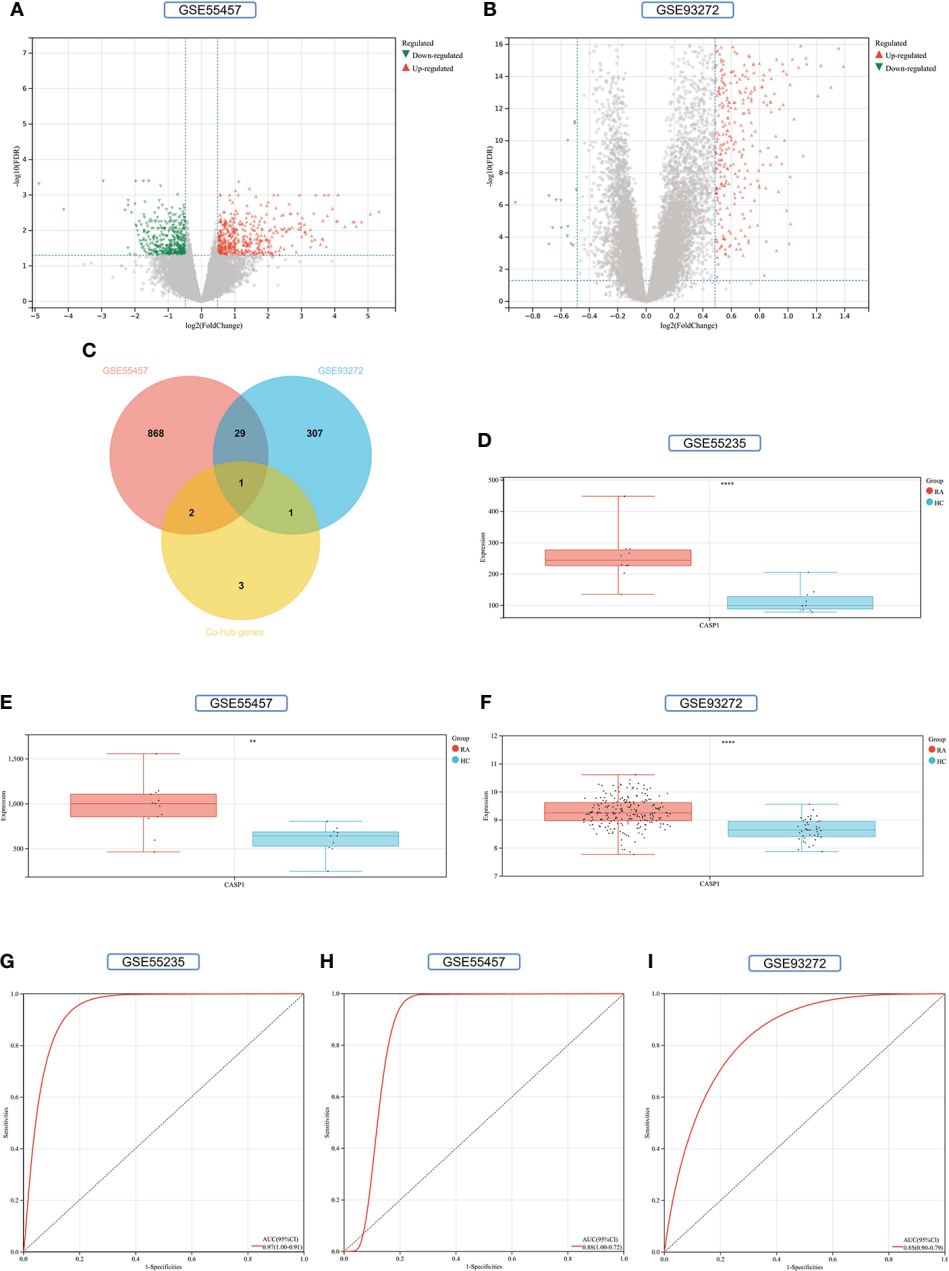
Screening and validation of key gene. **(A)** Volcano map of the GSE55457 dataset. **(B)** Volcano map of the GSE93272 dataset. Red triangles represent upregulated genes (*P* < 0.05), green triangles represent downregulated genes (*P* < 0.05), and gray dots represent genes not significantly differentially expressed across the RA and HC groups (*P* > 0.05). **(C)** Venn-diagram of RA-DEGs of GSE55457 and GSE93272 with Co-hub genes. **(D–F)** Expression of CASP1 in the GSE55235, GSE5457, and GSE93272 datasets, Red for the RA group and cyan for the HC group (**P < 0.01 and ****P < 0.0001). **(G–I)** The AUC of the ROC curve verifies the diagnostic validity of CASP1 in GSE55235, GSE55457和GSE93272 (*P* < 0.05).

900 DEGs were obtained from the GSE55457 validation set, of which 470 were upregulated genes and 430 were down-regulated genes (**Figure 11A**). 338 DEGs were obtained from the GSE93272 validation set, 322 upregulated genes, and 16 down-regulated genes (**Figure 11B**). The distribution of these two RA-DEGs was visualized separately using volcano plots. The only Key gene in the Venn-diagram intersection of the Co-hub genes with these two RA-DEGs is CASP1 (**Figure 11C**). CASP1 was highly expressed in the RA group in all three datasets (*P*<0.01) (**Figures 11D–F**). The AUC values of CASP1 in the GSE55235, GSE55457, and GSE93272 datasets were 0.97 (0.91-1.00), 0.88 (0.72-1.00), and 0.85 (0.79-0.90), respectively, all of which were greater than 0.8, using ROC curves to verify the diagnostic validity of CASP1 as a biomarker with good specificity and sensitivity (**Figures 11G–I**).

### Upstream pathway activity

**Figure 12:**
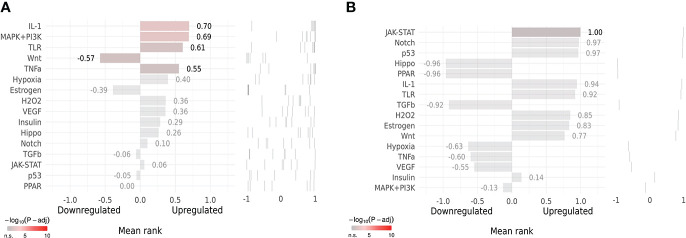
Upstream Pathway Activity. **(A, B)** SPEED2 platform analysis for Co-Hub Genes and key gene.

SPEED2 analysis in the context of all human gene sets showed that Co-Hub Genes were associated with the IL-1 signaling pathway (**Figure 12A**), and the Key gene (CASP1) was associated with the Janus kinase/signal transducer and activator of transcription (JAK-STAT) signaling pathway (**Figure 12B**).

### Immune infiltration analysis

**Figure 13:**
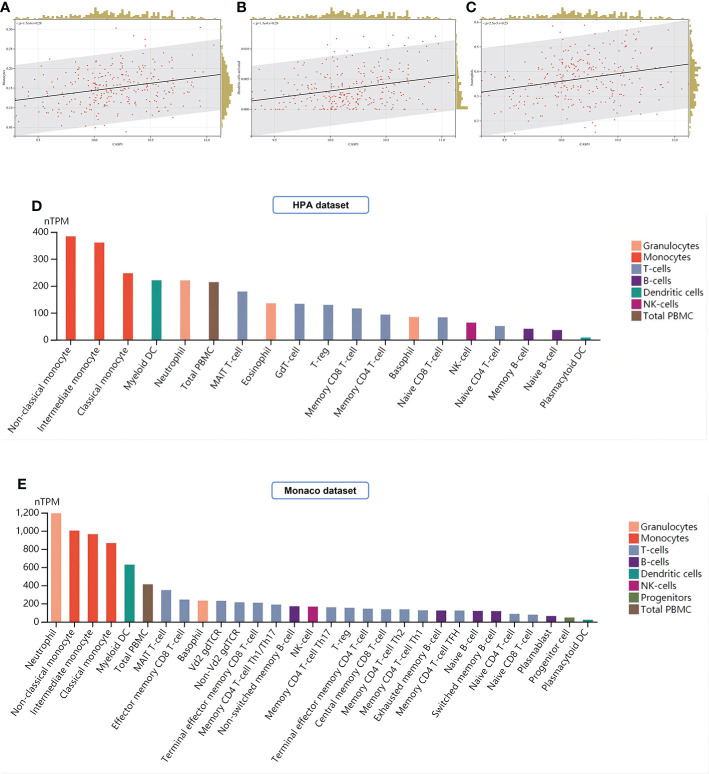
Analysis of immune cell infiltration. **(A–C)** Immune infiltrating cells positively associated with high CASP1 expression in LM22: Monocytes, Dendritic cells activated, and Neutrophils. **(D, E)** Distribution of CASP1 expression in immune cells from HPA datasets and Monaco datasets.

In this study, LM22 immune cell infiltration data in RA (GSE93272) was obtained by the CIBERSORT algorithm. CASP1 was positively correlated with monocytes, dendritic cells activated, and neutrophils by Pearson correlation coefficient analysis (**Figure 13A–C**). Both the HPA and Monaco datasets in the HPA platform showed that the top three immune cells with high CASP1 expression were monocytes, dendritic cells (DCs), and neutrophils (**Figures 13D, E**), thus validating our results for immune infiltration analysis.

### Molecular docking

**Figure 14:**
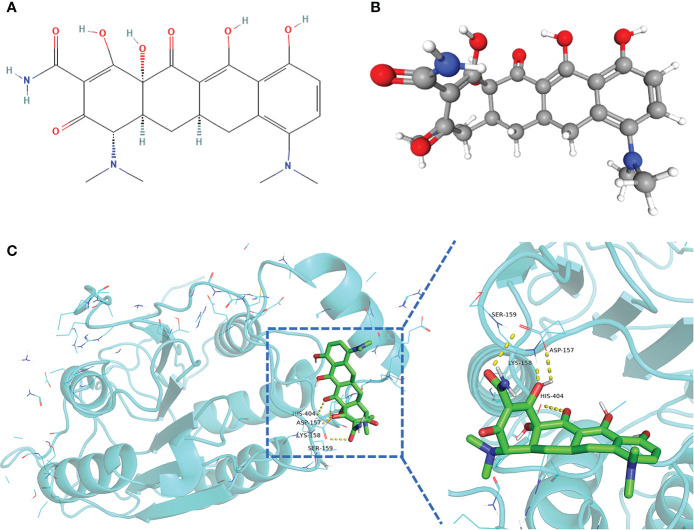
Structure of minocycline and molecular docking**. (A, B)** 2D and 3D structures of minocycline. **(C)** Results of molecular docking of minocycline with caspase-1 protein.

A drug’s conformation within a protein target binding site can be predicted by molecular docking, which can also predict the binding affinity. We obtained the 2D and 3D structures of minocycline (**Figures 14A, B**) and showed by MD analysis that minocycline forms four hydrogen bonds with the four amino acid residues ASP-157, LYS-158, SER-159, and HIS-404 of caspase-1, allowing minocycline to bind tightly to the active pocket of caspase-1 to form a stable complex (**Figure 14C**).

### Molecular dynamics simulation and MM-GBSA

**Figure 15:**
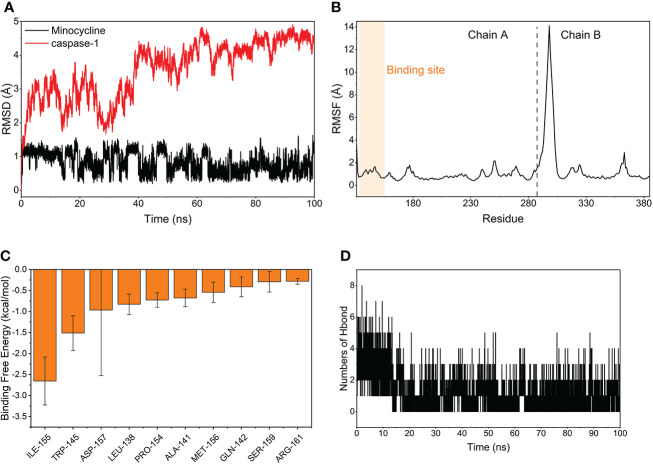
Molecular Dynamics Simulation and MM-GBSA. **(A)** Variation of the root means square deviation (RMSD) difference with time for small molecule compounds (black line) and proteins (red line) during molecular dynamics simulations. **(B)** Root mean square fluctuations (RMSF) are calculated based on molecular dynamics simulation trajectories. **(C)** The top 10 amino acids that contribute to small molecule and protein binding. **(D)** Changes in the number of hydrogen bonds between small molecules and proteins result from molecular dynamics simulations.

The MDS’s root-mean-square deviation (RMSD) depicts the movement of caspase-1 and minocycline; a greater value and amplitude of the RMSD suggests an intense movement and vice versa for a smooth movement. In **Figure 15A**, caspase-1 (red line) swings widely in the early portion of the simulation, begins to converge at 40 ns and plateaus later in the simulation, and caspase-1 fluctuates within 5Å overall, indicating that there has been no major disintegration. Minocycline’s (black line) value and amplitude were minor, fluctuating steadily around 1 Å and not reaching 1.5 Å. Typically, the RMSD of small molecules does not exceed 2 Å, indicating a weak conformational change. In conclusion, caspase-1 binds stably to the minocycline, almost tightly bound to the active site docked with caspase-1. The root-mean-square fluctuation (RMSF) indicates the flexibility of caspase-1 during the MDS process. When the drug attaches to the protein’s active site, its flexibility diminishes, stabilizing the protein and allowing the drug to have its biochemically active action. In **Figure 15B**, caspase-1 is composed of Chain A and Chain B. Overall, Chain A has a lower RMSF than Chain B, indicating that Chain A is less flexible. Minocycline interacts with Chain The start sequence of caspase-1 (the yellow background highlights the binding site) and the fact that the RMSF value for this region is less than 2 Å, indicating low protein flexibility, indicates that the binding of minocycline to caspase-1 is in a highly stable state.

Based on MDS, the binding energy of minocycline to caspase-1 was determined using MM-GBSA. It can reflect the binding pattern of the medication to the protein more precisely. A negative binding energy value (ΔG*_bind_*) implies that the medication binds to the protein with affinity, whereas a smaller value indicates a greater binding capability. The binding energy of minocycline/caspase-1 was -21.43 ± 3.89 kcal/mol, showing that minocycline has a strong binding affinity for caspase-1. The energy decomposition reveals that van der Waals and electrostatic forces are the primary contributors to their binding (**Table 3**). The amino acid residue decomposition results of MM-GBSA can be more accurate than the active amino acid residues obtained by molecular docking. In **Figure 15C**, The top 10 amino acids that play a key role in minocycline/caspase-1 were: ILE-155, TRP-145, ASP-157, LEU-154, ALA-141, MET-156, GLN-142, SER-159, ARG-161. The ILE-155 Δ*G_bind_* is -2.625 kcal/mol, TRP-145 is -1.513 kcal/mol, and ASP-157 is -0.967 kcal/mol (**Table 4**). Thus ILE-155, TRP-145, and ASP-157 are the major and maintained by hydrogen bonding minocycline/caspase-1 tightly bound amino acids. Hydrogen bonding is one of the greatest forces for the non-covalent binding of medicines and proteins, and an investigation of the number of hydrogen bonds is required to comprehend the relationship between minocycline and caspase-1. Based on MDS trajectory monitoring, we acquired the coordinates of the number of hydrogen bond formations between minocycline and caspase-1 over time. In **Figure 15D**, In the early part of the simulation (0-20 ns), the number of hydrogen bonds fluctuated in the range of 1-5, and in the middle and late part of the simulation (20-100 ns), the number of hydrogen bonds was mainly concentrated in 1-2. Thus, minocycline interaction with caspase-1 relies heavily on 1-2 hydrogen bonding forces.

**Table 3:** The prediction of binding free energies and energy components by MM/GBSA.

System name	Minocycline/caspase-1(kcal/mol)
**Δ*E*_vdw_**	-31.73±1.15
**Δ*E*_elec_**	-33.22 ±9.62
**ΔG_GB_**	47.07±5.66
**ΔG_surf_**	-3.55 ±0.11
**ΔG_bind_**	-21.43 ±3.89

ΔE_vdW_: van der Waals energy.

ΔE_elec_: electrostatic energy.

ΔG_GB_: electrostatic contribution to solvation.

ΔG_SA_: non-polar contribution to solvation.

ΔG_bind_: binding free energy.

**Table 4:** The binding energy of top10 amino acids contributes to minocycline/caspase-1 binding.

Residue	ΔG_bind_(kcal/mol)	STD
ILE-155	-2.6540984	0.571406151
TRP-145	-1.512980667	0.413772826
ASP-157	-0.966774667	1.55565844
LEU-138	-0.828761067	0.244439787
PRO-154	-0.727401867	0.173517763
ALA-141	-0.677654667	0.207741157
MET-156	-0.544312933	0.242333531
GLN-142	-0.412951867	0.235780676
SER-159	-0.292502533	0.244958839
ARG-161	-0.282	0.068203128

## Discussion

35 Co-genes were obtained by the intersection of COVID-19, RA (GSE55235), and pyroptosis-related genes enriched in NLR/TLR signaling pathway, NLRP3 inflammasome complex, death-inducing signaling complex, regulation of interleukin production and cytokine production involved in immune responses. The top 11 hub genes in Metascape were enriched in the network map of the SARS-CoV-2 signaling pathway, activation of the NLRP3 inflammasome by SARS-CoV-2, NLR signaling pathway, and interleukins signaling pathway. While they were enriched in GeneMANIA in inflammasome complex, IL production pathway, NF-κB signaling, TNF signaling, and regulation of cytokine-mediated signaling pathway. CASP1 was most involved in these enrichment pathways. Minocycline was found to be closely associated with CASP1 by NetworkAnalyst analysis. Therefore, based on bioinformatics analysis and further network pharmacology analysis, it was surprising to find that the 7 Co-hub genes obtained from the intersection of minocycline with COVID-19, RA (GSE55235), and pyroptosis were all contained in the top 11 hub genes of COVID-19, RA (GSE55235), and pyroptosis. One important TF (YY1) and two important miRNAs (hsa-mir-429 and hsa-mir-34a-5p) associated with CASP1 were obtained by TF-hub genes and miRNA-hub genes network. The key gene was validated by the GSE55457 and GSE93272 validation sets and obtained as CASP1, which was highly expressed in the RA group in all three datasets and validated with ROC for significantly good test performance. This gene coincided with the results of previous pathway analysis. SPEED2 analysis indicates that CASP1 is associated with the JAK-STAT signaling pathway. Immune cell infiltration analysis revealed that monocytes, dendritic cells activated, and neutrophils were able to express CASP1 at high levels, and the reliability of the results was verified by using the HPA dataset and Monaco dataset databases. Finally, the relationship between minocycline and caspase-1 was investigated and verified by MD, MDS, and MM-GBSA: minocycline can dock close to the active site of caspase-1 to form a highly stable state and exert the biochemical activity of the drug.

### Caspase-1 induces the classical pathway of pyroptosis

In this study, COVID-19, the crossover genes between RA and pyroptosis were enriched in the NLR/TLR signaling pathway, NLRP3 inflammasome complex, death-inducing signaling complex, regulation of interleukin production, NF-κB signaling, and TNF signaling. These pathways are all closely related to the caspase-1-induced pyroptosis pathway.

It is known that the innate immune system can recognize the viral pathogen-associated molecular pattern (PAMP) and host cell-derived damage-associated molecular pattern (DAMP) using the pathogen recognition receptor (PRR) (105–107). PRRs are divided into 2 main categories of 4 sensors: transmembrane proteins (TLRs, C-type lectin-receptors (CLRs)) and cytoplasmic proteins (RIG-I-like receptors (RLRs), NLRs) (108–110). NLRs, also known as versatile cytosolic sentinels (111, 112), play a significant role in the molecular processes (antigen presentation, inflammatory response, and cell death) linked to viral infectious diseases and autoimmune diseases (111, 113, 114). Five isoforms of NLRs, NLRA, NLRB, NLRC, NLRP, and NLRX1, activate two downstream signaling pathways: NOD1/NOD2 signaling and inflammasome signaling pathways (115), which recruit immune cells to produce pro-inflammatory cytokines (116). Caspases are a class of conserved cysteinyl proteases that activate themselves and other caspases by aspartate-specific cleavage (117) and can also cleave vast quantities of cellular substrates to drive cell death (e.g., apoptosis, pyroptosis) and inflammation (118). Caspases are classified as either apoptotic or inflammatory (119), with caspase-1 being the first member of the protease family of cysteases to be found (120) and the apical caspase of the inflammasome (121). caspase-1, one of the most typical inflammatory caspases, plays a crucial function in the regulation of pyroptosis and pro-inflammatory activities (122, 123). Since inflammatory caspases are inactive zymogens, they must be activated by the inflammasome to become proteolytically active (124). Inflammasomes are multiprotein complexes activated in response to endogenous and microbiological stimuli (125). The NLRP3 inflammasome is one of the most thoroughly researched and best-characterized inflammasomes in recent years (126), and it is the canonical activation platform for caspase-1 (127). The NLRP3 inflammasome is made up of a sensor (NLRP3), an adaptor (ASC), and an effector (caspase-1) (128). NLRP3 has a C-terminal Leucine rich repeat (LRR), a central nucleotide-binding and oligomerization domain (NACHT), and an N-terminal pyrin domain (PYD) (129, 130), whereas ASC has an N-terminal PYD and a C-terminal caspase recruitment domain (CARD) (131). full-length caspase-1 is composed of an N-terminal CARD, a main big catalytic domain (p20), and a C-terminal small catalytic subunit domain (p10) (132). PYD and CARD structural domains belong to the death domain (DD) fold superfamily (133).

NLRP3 inflammasome requires an initiation and activation pathway. The beginning step is the NF-κB-NLRP3 axis, in which the detection of PAMP/DAMP by a particular PRR (e.g., TLR) activates the NF-κB pathway, increasing NLRP3 expression (134, 135). During the initiation phase, phosphorylation and ubiquitination are further post-translational modifications of NLRP3 (136). The activation phase is the NLRP3/ASC/pro-caspase-1/caspase-1 axis, with NLRP3 recruiting the adaptor ASC through PYD-PYD interactions (137, 138), then ASC recruiting pro-Caspase-1 through CARD-CARD interactions (139, 140). Since autocatalytic activity permits autoconversion into p33 (both CARD and p20) and p10, removing CARD from the inflammasome after secondary autoconversion of caspase-1 p33/p10 releases an enzymatically active caspase-1 tetramer comprising p20/p10 subunits (141–143). There are two primary caspase-1 effector routes. One is the cleavage of pro-IL-1β and pro-IL-18 by the p20/p10 subunit of active caspase-1, which results in the release of IL-1β and IL-18 and the initiation of an inflammatory response (144–147). The second is for active caspase-1 to cleave and activate the executioner gasdermin D (GSDMD), cleave and remove its inhibitory GSDMD-C domain, and release the GSDMD-N domain (GSDMD-NT), allowing it to generate pores in the cell membrane and initiate pyroptosis (148–150).

Therefore, pyroptosis is a classical cytolytic type of PCD induced by caspase-1 (151). The pyroptosis pathway can be activated by various viral infections (64, 152–154) and can also be induced by autoantibodies to autoimmune diseases (AID) (155, 156). COVID-19 and RA share a tight relationship with the pyroptosis mechanism, which may be one of the pathogenic mechanisms by which COVID-19 interacts with RA to induce deterioration.

### Caspase-1 in COVID-19

In this study, the top 11 hub genes pathways of COVID-19, RA, and pyroptosis were enriched in the Network map of the SARS-CoV-2 signaling pathway, Activation of the NLRP3 inflammasome by SARS-CoV-2, IL, NF-κB, TNF signaling pathway and regulation of cytokine-mediated signaling pathway. Caspase-1 activation is not only a critical effector molecule in the development of acute respiratory distress syndrome (ARDS) (157, 158), but it is also a major contributor to the development of ALI (159, 160). In peripheral blood immune cells and tissues of COVID-19 patients, activated NLRP3 inflammasome, caspase-1, and high levels of GSDMD-NT were found, as well as elevated expression of IL-1β and IL-18 in serum (161–166). In animal investigations, high caspase-1 expression was also detected in the peripheral immune cells of SARS-CoV-2-infected rhesus monkeys (167). With the in-depth study of the mechanism of pyroptosis triggered by SARS-CoV-2, it was found that NSP6 in non-structural proteins (74, 168), N-protein (169), and S-protein (170) in structural proteins, and ORF3a protein (171) in auxiliary proteins all lead to overexpression and activation of NLRP3 inflammasome and caspase-1 and are positively correlated with the severity of COVID-19 (164). SARS-CoV-2 ultimately leads to an excessive inflammatory response in the form of a “cytokine storm” (172–174) and severe host cell pyroptosis (175). Cytokine storm is an uncontrolled, lethal immune disease characterized by the excessive release of pro-inflammatory cytokines and chemical mediators from immune cells (176, 177), capable of causing damage to multiple organs, including the respiratory system (165, 178), and it is believed to be a major cause of deterioration and death in COVID-19 patients (179).

In this study, immune cell infiltration analysis of COVID-19, RA, and the key gene for pyroptosis (CASP1) was found to be positively correlated with Monocytes, and the reliability of the results was verified by the HPA dataset and Monaco dataset databases. Among the numerous immune cells, monocytes play a vital part in the cytokine storm of COVID-19 patients (180). It was demonstrated that monocytes in COVID-19 patients are the outposts of SARS-CoV-2 invasion *via* TLR sensing and can release inflammatory cytokines by assembling NLRP3, activating caspase-1 to generate a “cytokine storm,” and synthesizing GSDMD-NT to induce cellular pyroptosis (72, 168). Monocytes from COVID-19 patients not only overexpress IL-1β and IL-18 but also show pyroptosis morphology, suggesting that pyroptosis is a possible key mechanism for cytokine storm in COVID-19 (123, 166).

### Caspase-1 in RA

The peripheral blood and synovial tissue of RA patients have been reported to contain a high level of expression and activation of the NLRP3 inflammasome and caspase-1, as well as a high level of expression of IL-1β and IL-18 (181–183). In animal investigations, inhibition of NLRP3 and caspase-1 was also found to be useful in alleviating the symptoms of arthritis in RA (CIA mouse model) (79). A cytokine network in the form of a cytokine storm, similar to that in COVID-19, is also present in RA and is a major factor in the disease’s onset, persistence, and progression (184, 185). The most important pro-inflammatory cytokines in RA are IL-1β and IL-18, and the expression of these cytokines is positively correlated with active disease status (186–188). In recent years the mechanism of pyroptosis has been shown to play a key role in the development of autoimmune diseases. In the course of the pro-inflammatory process, activation of the pyroptosis pathway causes host cells to release large amounts of pro-inflammatory cytokines and directs innate immune cells to the site of injury (119), which ultimately results in an overreactive immune response akin to a “cytokine storm” that sustains an ongoing autoimmune disease (189, 190).

In this study, immune cell infiltration analysis of the CASP1 in the RA dataset revealed that its expression was positively correlated with monocytes, dendritic cells activated, and neutrophils. It was found that high expression of NLRP3 and activated caspase-1 was detected in monocytes, dendritic cells, and neutrophils in the peripheral blood of RA patients, most notably in monocytes (181, 191, 192). Blood that circulates in the periphery Monocytes from RA patients can cleave GSDMD *via* the TLR4-NLRP3-caspase-1 pathway, resulting in pyroptosis and the production of a significant variety of cytokines, including IL-1β and IL-18, and are positively linked with disease activity (75, 193).

In conclusion, COVID-19 and RA are both capable of high expression of activated caspase-1 in peripheral blood and tissues. The invasion of SARS-CoV-2 in RA patients may enhance the caspase-1-induced pyroptosis mechanism, creating a vicious cycle of common outbreaks of “cytokine storm” and cell death, leading to increased hospitalization, morbidity, and mortality (194–197).

### The JAK-STAT pathway upstream of caspase-1

In this study, the functional enrichment of the collection of Co-genes and the Top 11 Hub Genes included the regulation of IL-6 production, and the Upstream Pathway of the key gene (CASP1) was closely related to the JAK-STAT signaling pathway. The JAK/STAT pathway, also called the IL-6 signaling pathway, can be activated by IL-6 (198, 199), which is also a significant indication of COVID-19 severity (1, 200). Activation of the JAK/STAT pathway, which produces pro-inflammatory cytokines, also a significant role in the development of rheumatoid arthritis (RA) (201). Thus the JAK/STAT pathway is also one of the crosstalk pathways of COVID-19 and RA (202, 203). JAK inhibitors, represented by Tofacitinib, have been approved by the FDA to treat moderately and severely active RA (204, 205). However, it increases the risk of viral infection (206, 207). Since IFN can trigger the JAK/STAT pathway to launch a cascade response against viral infection (208), JAK inhibitors would interfere with the natural IFN/ISG antiviral immune system in the context of SARS-CoV-2 infection. Currently, the WHO only advises baricitinib for the treatment of severe COVID-19 (209), and the evidence for the use of JAK inhibitors in the treatment of COVID-19 is weak and requires additional investigation (210–212). Since the JAK/STAT pathway can promote caspase-1 expression and activation *via* cytokines (e.g., GM-CSF) and interferons (e.g., IFN-γ) (213–216), this study, in conjunction with other evidence, suggests that the NLRP3/caspase-1 pathway is a key mechanism by which COVID-19 and RA disease exacerbate each other.

Therefore, we can look for drug targets downstream of the JAK/STAT pathway to avoid interfering with the IFN/ISG system by inhibiting the JAK/STAT pathway, but also to effectively inhibit the pyroptosis link, interrupting the “cytokine storm” that erupts from each other and thus interrupting the vicious cycle. Interestingly, caspase-1 is one of the common crosstalk targets between JAK/STAT and pyroptosis pathways.

### Minocycline and caspase-1

In the present COVID-19 pandemic, the discovery of new medications is challenging, time-consuming, risky, and less successful, and drug repurposing is a good option (217, 218). Minocycline is a second-generation semi-synthetic tetracycline derivative with a good safety profile (219). In addition to being a broad-spectrum antibiotic (220), it is also a broad-spectrum antiviral agent (e.g., HIV, WNV, DENV) (221–223) and possesses anti-inflammatory, antioxidant, anti-cell death (e.g., pyroptosis), immunomodulatory effects in terms of non-anti-microbial action (224–226). Fundamental investigations have demonstrated that minocycline inhibits caspase-1 activity in mice suffering from traumatic brain injury (TBI) (227); reduces the expression of caspase-1 to alleviate stress-induced depression in mice (228); acts as a caspase-1 inhibitor to delay the death of mice with Huntington’s disease (229); reduces caspase-1 activity in the retina of diabetic mice (230) and suppresses caspase-1 activation in mice with acute lung injury to reduce inflammation (231). Retrospective multicentre cohort studies have shown that minocycline inhibits caspase-1 to reduce the incidence of acute renal failure (232). In conclusion, minocycline can reduce IL-1β and IL-18 levels by selectively inhibiting caspase-1 expression and activation, and it can have anti-inflammatory and anti-pyroptosis effects in the lung and throughout the body. Minocycline could play an important potential role in treating patients with COVID-19 through these properties (233) and exert a powerful antimicrobial effect against co-infections/secondary bacterial infections in patients with COVID-19 (234, 235). A current clinical study indicates that the combination of minocycline and favipiravir has significant efficacy and safety in treating COVID-19 inpatients (236). Minocycline has also demonstrated efficacy in treating COVID-19 individuals who are secluded at home (237). In addition, minocycline has been known to be clearly and effectively used in treating RA for many years (238–240).

Thus, minocycline can counteract the “cytokine storm” inflammatory response and resist pyroptosis in patients with COVID-19 combined with RA by inhibiting the expression and activation of caspase-1. This process also indirectly demonstrates a potential caspase-1-directed pyroptosis and a shared pro-inflammatory mechanism between COVID-19 and RA, which requires further basic and clinical research.

## Conclusions

Bioinformatic analysis revealed that COVID-19, RA, and pyroptosis-related genes were enriched in pyroptosis and pro-inflammatory pathways (NLR/TLR signaling pathway, NLRP3 inflammasome complex, death-inducing signaling complex, regulation of interleukin production), natural immune pathways (activation of the NLRP3 inflammasome by SARS-CoV-2) and COVID-19-and RA-related cytokine storm pathways (IL, NF-κB, TNF signaling pathway and regulation of cytokine-mediated signaling). Of these, CASP1 is involved in most pathways. The genes related to minocycline were then obtained by network pharmacology analysis and intersected with COVID-19, RA, and pyroptosis to obtain the common hub gene, and then the key gene was verified as CASP1 by two validation sets. Caspase-1 may be an important mediator of the excessive inflammatory response induced by SARS-CoV-2 in RA patients through pyroptosis. Finally, minocycline was analyzed by computer-aided drug design as an effective drug against the mechanism of caspase-1-induced pyroptosis. Our study provides insight into the causes of the high hospitalization and mortality rates of COVID-19 combined with RA from a new perspective of pyroptosis and offers potentially effective drugs that could provide new directions for further analysis of its pathogenesis and the development of targeted clinical treatments.

## Data availability statement

The datasets presented in this study can be found in online repositories. The names of the repository/repositories and accession number(s) can be found in the article/**Supplementary Material**.

## Author contributions

QZ, RL and YC: Consulted the literature and prepared materials. QZ, RL, YC, QL, JZ and JBZ: Experimented and analyzed the data. QZ, RL and YC: Drawn up the manuscript. WW and WX devised the concept and supervised the study. All authors contributed to the article and approved the submitted version.

## Acknowledgments

We acknowledge the GEO and Genecards databases for providing their platforms and contributors for uploading meaningful datasets.

## Conflict of interest

The authors declare that the research was conducted in the absence of any commercial or financial relationships that could be construed as a potential conflict of interest.

## Publisher’s note

All claims expressed in this article are solely those of the authors and do not necessarily represent those of their affiliated organizations, or those of the publisher, the editors and the reviewers. Any product that may be evaluated in this article, or claim that may be made by its manufacturer, is not guaranteed or endorsed by the publisher.

## Glossary

**Table:**

Go	Gene Ontology
KEGG	Kyoto Encyclopedia of Genes and Genomes
PPI	Protein-Protein Interaction
TF	Transcription Factor
PCA	Principal Component Analysis
ROC	Receiver Operating Curve
MM-GBSA	Molecular Mechanics/Generalized Born Surface Area
DEG	Differentially Expressed Genes
GEO	Gene Expression Omnibus
BioGRID	the Biological General Repository for Interaction Datasets
BP	Biological Process
CC	Cellular Component
MF	Molecular Function
PRR	Pathogen Recognition Receptor
NLR	NOD-Like Receptor
TLR	Toll-Like Receptor
CLR	C-Type Lectin-Receptor
RLR	RIG-I-Like Receptor
NLRP3	The NOD-Like Receptor Family Pyrin Domain Containing 3
ASC	Apoptosis-Associated Speck-Like Protein
DD	Death Domain
PAMP	Pathogen-Associated Molecular Pattern
DAMP	Damage-Associated Molecular Pattern
RCD	Regulated Cell Death
ISG	Interferon-stimulated Gene
HC	Healthy Controls
NOD	Nucleotide-Binding Oligomerization Domain
AUC	Area Under the Curve
JAK	Janus Kinase
STAT	Signal Transducer and Activator of Transcription
DCs	Dendritic cells
RMSD	Root-Mean-Square Deviation
RMSF	Root-Mean-Square Fluctuation
NF-Kb	Nuclear Factor-kappa B
AID	Autoimmune Disease
NSP6	Non-Structural Protein 6
N-protein	Nucleocapsid protein
S-protein	Spike protein
E-protein	Envelope protein
M-protein	Membrane protein
LRR	Leucine Rich Repeat
NACHT	Nucleotide-Binding and Oligomerization Domain
PYD	Pyrin Domain
CARD	Caspase Recruitment Domain
ARDS	Acute Respiratory Distress Syndrome
