# Supplementary material for: SARS-CoV-2 induces “cytokine storm” hyperinflammatory responses in RA patients through pyroptosis
Source: Front Immunol. 2022 Dec 1;13:1058884. doi: 10.3389/fimmu.2022.1058884 (PMC9751040; doi:10.3389/fimmu.2022.1058884)
Supplement: Supplementary file 1 [file Table_1.docx]

**Supplementary Table 1.** COVID-19-associated genes in the relevant reference.

| Reference | Tissue(Homo sapiens) | Experiment type | Gene ID |
| --- | --- | --- | --- |
| Xiong et al., 2020(94) | Peripheral blood mononuclear cells, Bronchoalveolar lavage fluid | Array | IL 33, IL18, IL10,CXCL1, CXCL2, CXCL6, CXCL8,CXCL10, CXCL10/IP-10,CCL2/MCP-1,CCL3/MIP-1A, CCL4/MIP1B,TNFSF10, TIMP1, C5, AREG, NRG1, ADA2, HK1, GAT1,PGD, PLA2G15, CTSD, GAA, LAIR1 |
| Ziegler et al., 2020(92) | Nasal polyps, Lung lobe, ethmoid sinus surgical tissue, Ileum | Array | STAT1,IFI6,IFNAR1, IFNGR2,GBP2,IFITM1,TRIM27, NT5DC1, ARL6IP1,TMPRSS2, ACE2, TRIM28, APOA1, FABP6, ENPEP, FI35, XAF1 |
| Jain et al., 2020(93) | Nasopharyngeal swabs | Array | IL6, IL10, IL11, IL19, IL3RA,IL21RA,IL18R1,IFI44,IFIT1,IFIT1B, IFIH1,CXCL5, CXCL12, CCL2, CCL4, CXCL10,CSF2, TNFSF11, TNFRSF11B, BMP2, BMP7, PDGFA,C4BPA, CCR6, CCR22, CCR25, SERPINE1, SERPINF2 |
