# Supplementary material for: SARS-CoV-2 induces “cytokine storm” hyperinflammatory responses in RA patients through pyroptosis
Source: Front Immunol. 2022 Dec 1;13:1058884. doi: 10.3389/fimmu.2022.1058884 (PMC9751040; doi:10.3389/fimmu.2022.1058884)
Supplement: Supplementary file 2 [file Table_2.docx]

**Supplementary Table 2.** Minocycline-related target genes in 4 databases.

| [Database](javascript:;) | Minocycline-related genes |
| --- | --- |
| SwissTargetPrediction | GRK6, MMP13, MMP2, MMP9, TDP1, ESR2, CDK1, CHEK2, CHEK1, CCND1 CDK4, CDK1 CCNB1, CCNE2 CDK2 CCNE1, CDK2, EGLN1, CDK4, CDK2 CCNA1 CCNA2, CNOT7, ADRB2, ADRB1, DRD3, LRRK2, GRK7, HIPK4,  TAOK2, MAK, CDKL5, ICK,VRK2, PIP4K2C, CDK13, CSF1R, ABL1, CDK5R1 CDK5, CCNE1 CDK2, PIP5K1C, DSTYK, KIT, FLT3, PDGFRA,  EGFR, EPHA2, CCNB3 CDK1 CCNB1 CCNB2, CCND3 CCND1 CDK4 CCND2, MAP2K3, CDK7 CCNH, CDK9 CCNT1, PIM1, JAK3, MAPK8,  DYRK1A, RPS6KA3, PHKG2, CSNK1G1, CAMK4, CDK6, RPS6KA1, PRKD3,  GSK3B, MAPK10, CSNK1A1, CAMK2D, GSK3A, MAP3K9, MAP3K10,  MAP2K4, JAK2, ROCK2, PRKCD, PRKCA, ERBB4, CDK7, CDK9, RPS6KA4, ROCK1, IRAK1, PKN1, PYGM, IKBKE, TYK2, PRKCE, PRKCH, IRAK4, PLK4, CAMK2G, AAK1, PRKD1, RPS6KA2, PRKCQ, STK17B, EPHA5, PHKG1, CDK5, PRKAA1, CAMK2B, CAMK2A, MKNK2, FRK, CLK1, CLK2, CLK3 |
| CTD | CASP1, CASP3, BCL2, NOS2, TNF, HTT, IL1B, MMP2, CYCS, MMP9, HIF1A, APP, CASP12, CASP9, IL6, TGFB2, AIFM1, BAX, BID, CASP8, DIABLO, EGLN1, EIF2S1, IFNG, IL10, VHL, XIAP, CASP7, CCL2, CDKN1B, CXCL8, ENO1, IL2, MMP13, PRDX3, STMN1, TGFB1, AIF1, ATF3, ATF6, ATP5A1, ATP5H, BACE1, BAK1, CCL3, CCL4, CCL5, CHIL3, COX5A, DDIT3, DLST, EIF2AK2, EIF2AK3, ERN1, FMR1, GJC2, GRIA1, GRIN2A, GRIN2C, GSK3B, HLA-A, HLA-B, HLA-F, HSPA5, HSPD1, ICAM1, IL6R, INA, JUN, MAPK8, MAPKAPK5, MMP3, MR1, PAFAH1B2, PRDX2, PRKN, PRTN3, PTGS1, RELA, SLC22A6, SLC22A7, SLC2A1, SLC6A3, SNAP25, SOD2, TP53, TPO, TUBA1A, VEGFA, VEGFC, VEGFD, XBP1 |
| Drugbank | CASP1, CASP3, IL1B, RPSL, RPSD, 16S rRNA, ALOX5, MMP9, VEGFA, CYCS, "Mitogen-activated Protein Kinases", NOS2 |
| STITCH | CASP1, CASP3, IL6, MMP9, VEGFA, MMP2, TNF, IDO1, MMP12, HAVCR2 |
